# Survival outcomes of lung metastases from colorectal cancer treated with pulmonary metastasectomy or modern systemic chemotherapy: a single institution experience

**DOI:** 10.1186/s13019-023-02434-8

**Published:** 2023-11-14

**Authors:** Yutaka Shishido, Masayuki Ishii, Tetsuo Maeda, Yujiro Kokado, Daiki Masuya, Toshiyuki Kusama, Koji Fujimoto, Hiroshi Higashiyama

**Affiliations:** 1 Department of Gastrointestinal Surgery, Shinko Hospital, 1-4-47, Wakinohama-cho, Chuo-ku, Kobe, 6510072 Hyogo Japan; 2 Department of Thoracic Surgery, Shinko Hospital, 1-4-47, Wakinohama-cho, Chuo-ku, Kobe, 6510072 Hyogo Japan; 3 Department of Medical Oncology, Shinko Hospital, 1-4-47, Wakinohama-cho, Chuo-ku, Kobe, 6510072 Hyogo Japan

**Keywords:** Colorectal cancer, Metastasis, Lung, Surgery, Chemotherapy

## Abstract

**Background:**

Although pulmonary metastasectomy is an accepted treatment strategy for resectable lung metastases (LM) from colorectal cancer (CRC), its survival benefits are controversial. In contrast, recent advancements in chemotherapy have significantly improved metastatic CRC prognosis. This study aimed to evaluate survival outcome of LM from CRC in the age of newly developed chemotherapy.

**Methods:**

We retrospectively reviewed 50 patients who underwent complete resection and 22 patients who received chemotherapy as definitive treatment for LM from resected CRC at our hospital. The present study was limited to patients who started treatment for isolated LM after molecular targeted drugs became available in Japan.

**Results:**

Overall survival (OS), cancer-specific survival (CSS), disease-free survival (DFS) rates after pulmonary resection were 64.5%, 66.4%, and 32.6% at five years, respectively. OS and CSS rates of chemotherapy patients were 26.8% and 28.3% at five years, with a median progression-free survival time of 10.0 months. When compared the characteristics of surgical and chemotherapy patients, patients with pN factors of CRC (p = 0.013), smaller size (p < 0.001), larger number (p < 0.001), and bilateral (p < 0.001) LM received chemotherapy. Univariate analysis showed that multiple LM and rectal lesions were poor prognostic factors for OS (p = 0.012) and DFS (p = 0.017) in surgical patients, and rectal lesions were a poor prognostic factor for OS (p = 0.013) in chemotherapy patients.

**Conclusions:**

Pulmonary metastasectomy showed a favorable survival in patients with LM from CRC. Despite the high recurrence rate after metastasectomy and recent advances in chemotherapy, surgical resection could still be considered as a valid option among multidisciplinary treatments.

**Trial registration:**

The research plan was approved by the Institutional Review Board of Shinko Hospital (No. 2142) on February 7, 2022.

## Main text

### Background

A considerable proportion of patients with colorectal cancer (CRC) have metastatic tumors at the time of diagnosis or after resection of the primary tumor [1], with the lung being one of the most commonly involved organs [2]. Pulmonary metastasectomy is an accepted treatment strategy for resectable lung metastases (LM) from CRC, and this is supported by previous retrospective studies that show a 5-year survival of 27–68% [3]. However, most patients developed disease recurrence after metastasectomy, and recurrence rates in patients with poor prognostic factors were over 60% [4–6]. Due to the postoperative high recurrence rate, a substantial portion of the patients required systemic chemotherapy for the tumor recurrence after metastasectomy [7]. Thus, systemic chemotherapy after the recurrence could play an important role to improve survival of patients with LM from CRC. Recent advancements in chemotherapy have significantly improved the prognosis of unresectable metastatic CRC [8–12], and several studies also suggest that chemotherapy would contribute to an improvement of survival after metastasectomy for LM from CRC [13, 14]. However, few articles have investigated the impact of newly developed drugs, such as molecular targeted drugs (MTDs), on postoperative outcomes of LM. Therefore, it would be meaningful to investigate survival outcomes of pulmonary metastasectomy performed after the introduction of MTDs.

There is a general assumption among surgeons and oncologists that the chance of long-term survival for patients with LM from CRC would be near zero without metastasectomy [15]. Moreover, the pulmonary metastasectomy can be performed safely with low morbidity and mortality [4, 14]. As a result, this treatment approach is spreading more widely. However, there is no conclusive evidence that metastasectomy improves survival in LM from CRC, and a recent randomized controlled trial had to be stopped early due to slow enrollment and did not have sufficient power to demonstrate a 10% improvement in 5-year survival compared with active surveillance [7]. Given the unclear benefit of metastasectomy and the development of chemotherapy, it would be important to evaluate the survival outcome of surgery and modern systemic chemotherapy.

In this study, we retrospectively reviewed the data of patients who underwent pulmonary metastasectomy or received modern chemotherapy alone for LM from CRC at our hospital, and investigated patient’s characteristics and survival outcomes to evaluate treatment effect for isolated LM in the age of newly developed chemotherapy.

## Methods

### Study design

This study was a single-center, retrospective observational study, evaluating the survival outcomes of pulmonary metastasectomy and modern chemotherapy in patients with isolated LM from CRC. We reviewed data of patients who were treated for LM from CRC at Shinko Hospital between January 2010 and January 2021, considering that MTDs such as bevacizumab (BEV), an anti-vascular endothelial growth factor (VEGF) antibody drug, and cetuximab, an anti-epidermal growth factor receptor (EGFR) antibody drug, had officially become available for clinical use in Japan by 2010. At our hospital, the criteria for metastasectomy for LM were basically based on the National Comprehensive Cancer Network (NCCN) guidelines [16], which are: The primary CRC is controlled, pulmonary lesions are resectable, the patient can tolerate pulmonary resection, and there are no unresectable extrapulmonary lesions. When patients did not meet these criteria, they received systemic chemotherapy for LM. After starting the treatment for LM from CRC, patients’ therapeutic plans are determined based on discussions between colorectal surgeons and medical oncologists. The research plan was approved by the Institutional Review Board of Shinko Hospital (No. 2142) on February 7, 2022. Due to the retrospective nature of this study using unidentifiable data, informed consent was obtained in the form of opt-out on the web-site, so that all patients could reject to participate in this study anytime.

### Patients

A total of 148 patients who were treated for LM from CRC at our hospital were eligible for inclusion. We excluded 74 patients with unresectable extrapulmonary lesions and/or an uncontrollable primary tumor at the initiation of treatment for LM in order to evaluate the treatment outcome of isolated LM. We also excluded one patient who chose best supportive care and one patient who had no available data in the medical record. Ultimately, 72 patients were included in this retrospective study. Five surgical patients and one chemotherapy patient, all with synchronous CRCs, were excluded from univariate analyses and characteristic analyses of CRC data, because synchronous CRCs had several different sets of CRC data per patient, which created difficulty in conducting these analyses.

### Data collection

Clinicopathological information about patients, primary CRC, and LM was obtained from medical records. After resection of the primary tumor or LM, the follow-up was conducted according to the NCCN guidelines. LM was diagnosed with chest computed tomography (CT) by multiple doctors including radiologists. The date of pulmonary recurrence was recorded as the date when LM was first detected on chest CT by retrospective review. When patients had LM at the time of primary tumor resection, we defined LM as synchronous LM, and the date of pulmonary recurrence was recorded as the date of colorectal surgery. The disease-free interval (DFI) was calculated from the date of colorectal surgery to the date of pulmonary recurrence. If patients had synchronous LM, the DFI was recorded as 0. In cases of multiple LM, the diameter of the largest tumor was used as the metastatic tumor size. In patients treated with chemotherapy, tumor markers were measured monthly and CT scans were done every 3–4 months to evaluate disease progression during the treatment. The response assessment of LM to chemotherapy was performed according to the Response Evaluation Criteria in Solid Tumors version 1.1 [17]. Adverse events were categorized according to the National Cancer Institute Common Terminology Criteria for Adverse Events version 5.0 [18].

### Outcomes

The primary outcome was 5-year overall survival (OS), cancer-specific survival (CSS), and disease-free survival (DFS) in patients treated with pulmonary metastasectomy. The secondary outcome was poor prognostic factors for pulmonary metastasectomy, and survival difference between surgery and chemotherapy. We also investigated the distribution of patient’s characteristics in surgery and chemotherapy groups to better understand each background.

### Statistical analysis

The 5-year OS, CSS, DFS, and progression-free survival (PFS) were assessed using the Kaplan-Meier method, and univariate analyses were performed using the log-rank test. The distribution of characteristics between surgical and chemotherapy patients was evaluated using Fisher’s exact test for categorical variables and the Student’s t-test for numerical variables. All data were analyzed statistically using EZR (Saitama Medical Center, Jichi Medical University, Saitama, Japan), which is a graphical user interface for R (The R Foundation for Statistical Computing, Vienna, Austria) [19]. All P values were two-sided, and differences were considered statistically significant at P < 0.05.

## Results

### Patient characteristics

Of the 72 patients who had LM from resected CRC, 50 underwent pathologically complete resection and 22 received definitive systemic chemotherapy. In patients treated with pulmonary metastasectomy, lobectomy and sub-lobar resection were performed in 17 and 33 patients, respectively. Of the 22 chemotherapy patients, 14 were not eligible for pulmonary metastasectomy because of unresectable LM; three patients requested chemotherapy rather than surgery; two were scheduled to undergo two-stage surgery for bilateral LM, but became ineligible for secondary surgery due to tumor progression; one patient received chemotherapy considering respiratory dysfunction after lung resection; and no clear reason was found in the medical records for the other two patients.

Clinicopathological data of the 72 patients are shown in Table 1. Preoperative chemoradiation therapy for the primary tumor was conducted in only one patient who was treated with metastasectomy. In patients treated with pulmonary metastasectomy, complete extrapulmonary resections were performed in five patients with liver metastases and two patients with pelvic recurrence before lung resection, and one patient with liver metastases after lung resection. In patients whose LM were treated with chemotherapy alone, hepatectomy was performed in five patients before the start of chemotherapy administration, and in one patient after the start of chemotherapy. When the characteristics of the surgical and chemotherapy patients were compared, patients with pN factors of CRC (p = 0.013), smaller LM size (p < 0.001), larger LM number (p < 0.001), and bilateral LM (p < 0.001) received systemic chemotherapy (Table 1).

**Table 1:** Patient characteristics and the distribution between surgery and chemotherapy

Characteristics		All patients (%)	Surgery (%)	Chemotherapy (%)	*p* value
Age (years)	Median [range]	69 [37–89]	69 [37–89]	70 [51–82]	0.372
Sex	Male	42 (58.3)	30 (60)	12 (54.4)	0.796
	Female	30 (41.7)	20 (40)	10 (45.5)	
Extrapulmonary metastasectomy	No	58 (80.6)	42 (84)	16 (72.7)	0.335
	Yes	14 (19.4)	8 (16)	6 (27.3)	
Perioperative chemotherapy for CRC	No	44 (61.1)	27 (54)	17 (77.3)	0.072
	Yes	28 (38.9)	23 (46)	5 (22.7)	
CRC location	Colon	40 (60.6)	29 (64.4)	11 (52.4)	0.422
	Rectum	26 (39.4)	16 (35.6)	10 (47.6)	
CRC tumor size (mm)	Mean [range]	47 [18–110]	46 [18–110]	47 [20–100]	0.877
pT classification	T0	1 (1.5)	1 (2.2)	0 (0)	0.946
	T1	3 (4.5)	2 (4.4)	1 (4.8)	
	T2	13 (19.7)	9 (20.0)	4 (19.0)	
	T3	30 (45.5)	19 (42.2)	11 (52.4)	
	T4	19 (28.8)	14 (31.1)	5 (23.8)	
pN classification	N0	24 (36.4)	21 (46.7)	3 (14.3)	0.013
	N1	25 (37.8)	16 (35.6)	9 (42.9)	
	N2	15 (22.7)	8 (17.8)	7 (33.3)	
	N3	2 (3.0)	0 (0)	2 (9.5)	
CRC lymphatic invasion	Absent	10 (15.2)	9 (20)	1 (4.8)	0.15
	Present	56 (84.8)	36 (80)	20 (95.2)	
CRC vascular invasion	Absent	23 (34.8)	15 (33.3)	8 (38.1)	0.784
	Present	43 (65.2)	30 (66.7)	13 (61.9)	
Synchronicity of LM	No	36 (50)	28 (56)	8 (36.4)	0.2
	Yes	36 (50)	22 (44)	14 (63.6)	
DFI (months)	Mean [range]	10.3 [0-57.1]	11.7 [0-57.1]	6.8 [0-44.8]	0.179
LM size (mm)	Mean [range]	15 [2–55]	17 [2–55]	8 [2–12]	< 0.001
LM number	Mean [range]	2.8 [1–14]	1.6 [1–6]	5.4 [1–14]	< 0.001
Bilaterality of LM	Hemilateral	49 (68.1)	44 (88)	5 (22.7)	< 0.001
	Bilateral	23 (31.9)	6 (12)	17 (77.3)	
Pretreatment CEA > 5ng/ml	No	47 (65.3)	33 (66)	14 (63.6)	1
	Yes	25 (34.7)	17 (34)	8 (36.4)	

CRC: colorectal cancer, LM: lung metastases, DFI: disease-free interval, CEA: carcinoembryonic antigen

### Survival outcomes

**Fig. 1:**
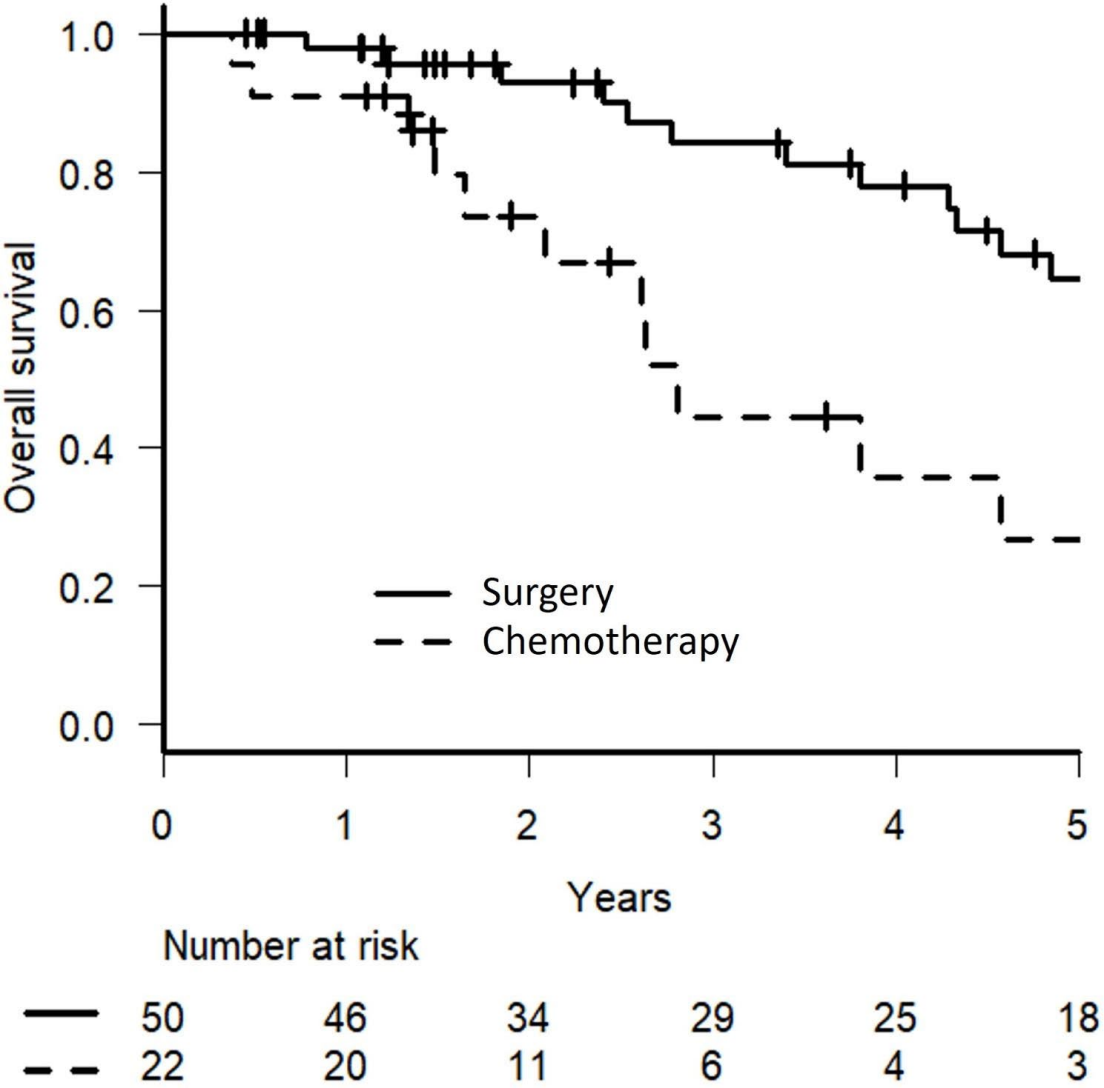
Kaplan-Meier curves of overall survival in patients treated with surgery or chemotherapy for lung metastases from colorectal cancer

**Fig. 2:**
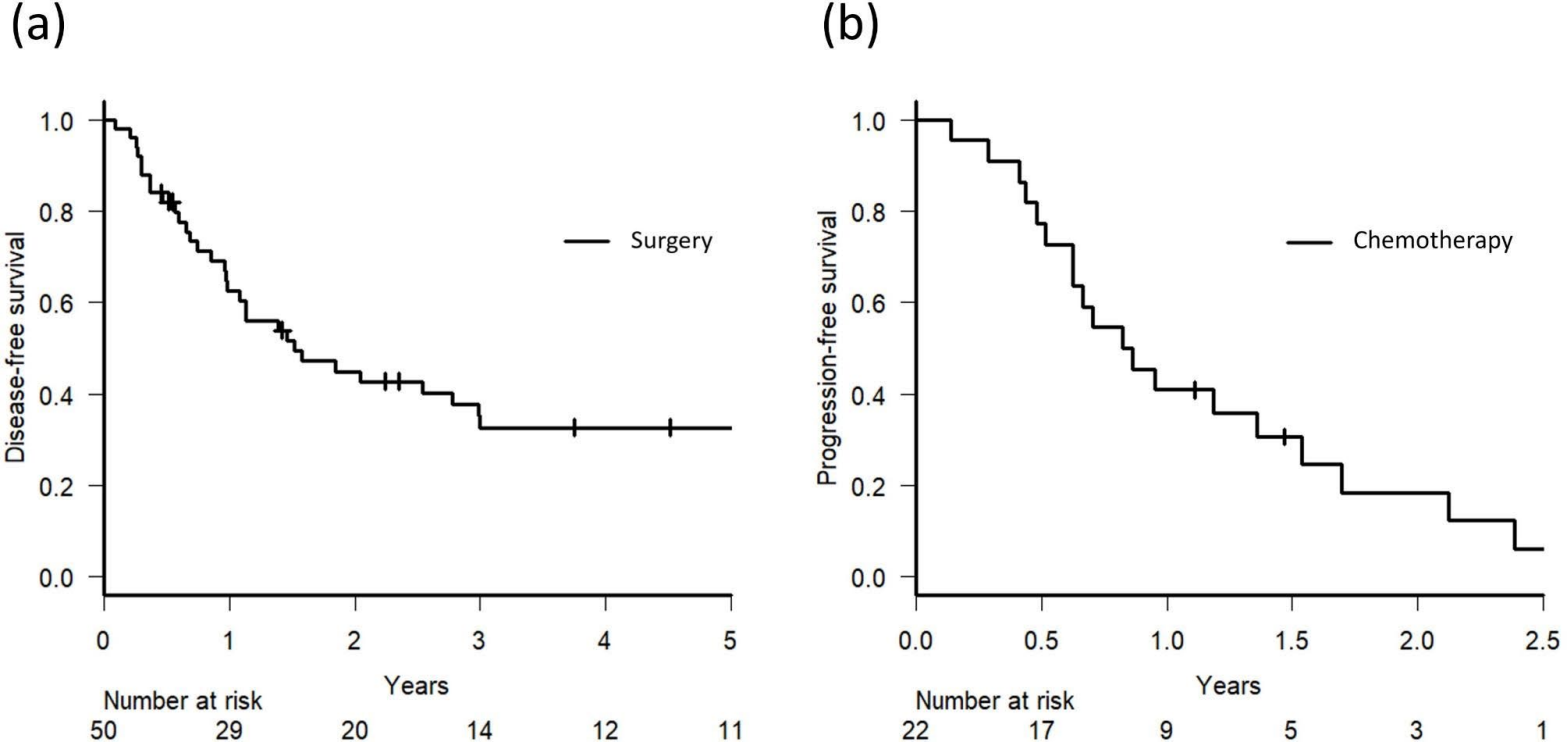
**(a)** Kaplan-Meier curve of disease-free survival in patients who underwent surgery for lung metastases from colorectal cancer **(b)** Kaplan-Meier curve of progression-free survival in patients who received chemotherapy for lung metastases from colorectal cancer

The median follow-up time was 3.9 (range: 0.5– 11.3) years in surgical patients and 2.0 (range: 0.4–6.4) years in chemotherapy patients. The 5-year OS, CSS, and DFS rates of surgery patients were 64.5% (Figs. 1), 66.4%, and 32.6% (Fig. 2a), respectively. In chemotherapy patients, the 5-year OS rate, median OS duration, CSS, and 5-year PFS were 26.8% (Figs. 1), 24.3 (range: 4.5–79.3) months, 28.3%, and 10.0 (range: 1.7–33.8) months (Fig. 2b), respectively.

Of the 50 surgical patients, 29 had tumor recurrence after pulmonary metastasectomy, and 14 died during the follow-up period. Tumor recurrence occurred in the lung or thoracic lymph nodes in 14 patients, the lung and liver in four patients, the liver in three patients, pelvic lesions in three patients, the brain in two patients, and other sites in three patients. Of the 29 patients, 17 received systemic chemotherapy for tumor recurrence. The chemotherapy regimens were decided based on the patient’s anti-EGFR and anti-RAS antibody status and medical conditions: 4 patients received BEV/Irinotecan (IRT)/Fluorouracil (5-FU) as first-line chemotherapy, 3 patients received BEV/Platinum (PT)/5-FU, 3 patients received panitumumab (PMAB)/PT/5-FU, 2 patients received PT/5-FU, 2 patients received IRT/5-FU, 2 patients received PMAB/IRT/5-FU, and one PMAB/IRT. Repeated pulmonary resections were performed in eight patients. No perioperative death was associated with surgical complications. Of the eight patients, six patients did not have a second tumor recurrence without systemic chemotherapy after repeated surgery.

Of the 22 chemotherapy patients, seven patients received BEV/PT/5-FU as the first-line of chemotherapy for LM; six received PT/5-FU; two received BEV/IRT/5-FU; two received PMAB/PT/5-FU; two received 5-FU; one received BEV/PT/5-FU; one received PMAB/IRT/5-FU; one received PMAB/IRT. During the course of chemotherapy, 20 patients were regarded as having progressive disease, including 17 patients with exacerbations of LM, and 14 patients died. There were no chemotherapy-related deaths. Grade 3–4 adverse events occurred in 12 patients: Neutropenia in seven, diarrhea in two, and skin reaction in two. Of the three patients who received chemotherapy for resectable LM at the patient’s request, two patients died of cancer-specific death and one patient had progressive disease, with a median OS of 16.6 months. In those who received systemic chemotherapy, none turned to be eligible for pulmonary metastasectomy.

**Fig. 3:**
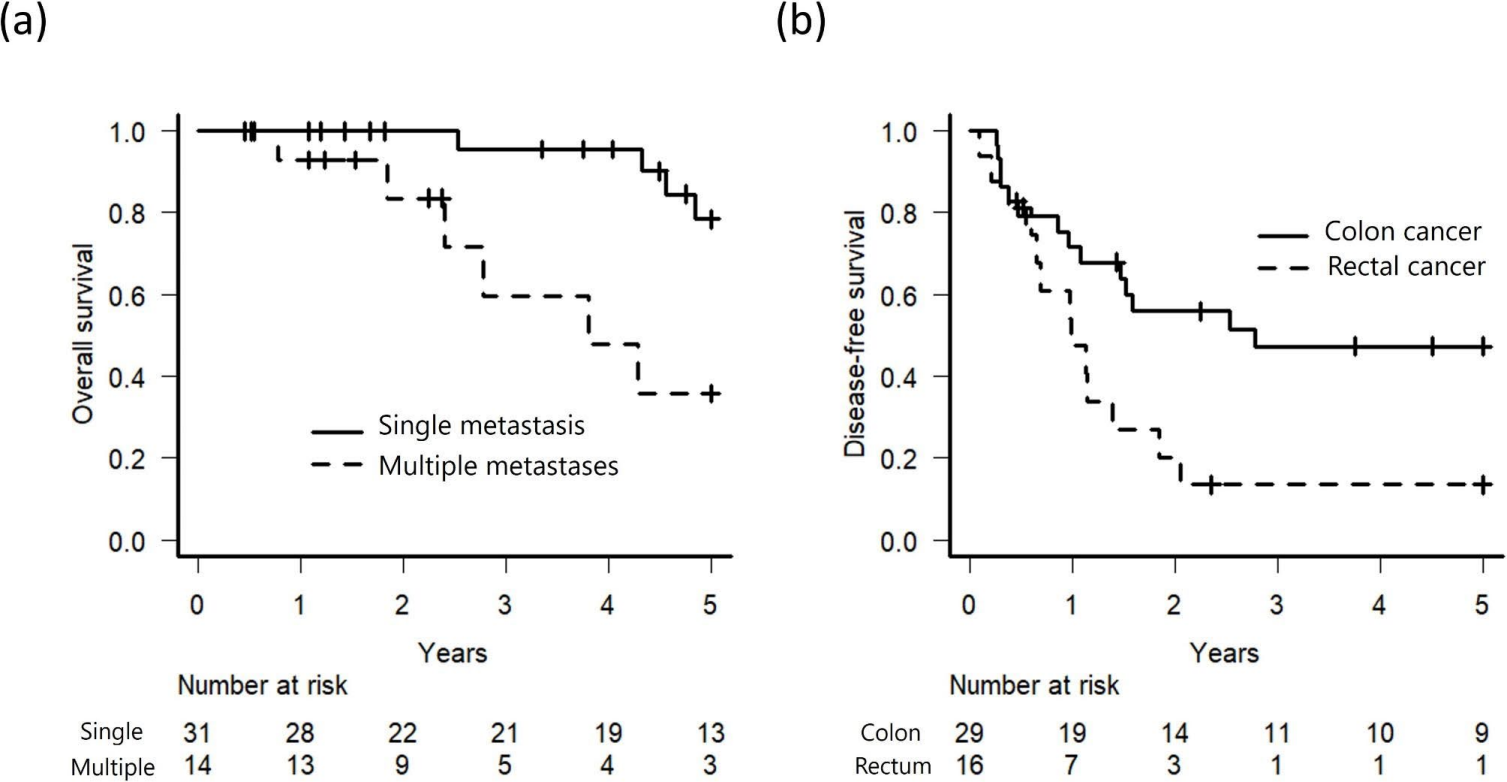
**(a)** Kaplan-Meier curves of overall survival in patients who underwent surgery for single or multiple lung metastases **(b)** Kaplan-Meier curves of disease-free survival in patients who underwent surgery for lung metastases from colon cancer or rectal cancer

The results of the univariate analysis for long-term prognosis are shown in Table 2. In surgical patients, multiple LM (p = 0.012) were a poor prognostic factor for 5-year OS (Fig. 3a), and rectal cancer (p = 0.017) predicted a poor prognosis for 5-year DFS (Fig. 3b). In surgical patients who had tumor recurrence after pulmonary metastasectomy, the median time from surgery to tumor recurrence was 311 days. When we analyzed the prognostic impact of early tumor recurrence (tumor recurrence within 311 days after surgery) using univariate analysis, there was no significant difference in OS between patients with early and late recurrence. In chemotherapy patients, rectal cancer (p = 0.013) predicted a poor prognosis for 5-year OS.

**Table 2:** Univariate analysis of prognostic factors associated with disease-free survival and overall survival

Treatment	Surgery (n = 50)		Chemotherapy (n = 22)
	DFS rate (%)	OS rate (%)	OS rate (%)
Characteristic, factor	Factor /No factor (*p* value)	Factor /No factor (*p* value)	Factor /No factor (*p* value)
Age, > 70 y	53/67 (0.668)	48/78 (0.100)	0/34 (0.544)
Sex, male	33/34 (0.580)	58/70 (0.943)	22/34 (0.286)
Extrapulmonary metastasectomy	19/35 (0.824)	63/65 (0.453)	27/26 (0.434)
CRC location, rectum	14/47 (0.017)	53/71 (0.236)	0/50 (0.013)
CRC tumor size, > 40 mm	29/42 (0.241)	54/77 (0.232)	18/32 (0.099)
CRC pT factor, 4/3/2/1/0	32/43/13/100 (0.127)	55/78/50/100 (0.565)	0/28/67/0 (0.37)
CRC pN factor, 1–3/0	28/41 (0.171)	56/76 (0.244)	20/50 (0.080)
CRC pStage, IV/III/II/I	36/31/45/29 (0.726)	63/86/67/44 (0.404)	-
Synchronicity of PM	29/35 (0.639)	55/70 (0.150)	40/0 (0.608)
DFI, < 1 year	33/33 (0.584)	62/69 (0.669)	30/31 (0.64)
LM size, > 20 mm	46/27 (0.135)	61/67 (0.628)	-
LM size, > 10 mm	-	-	0/36 (0.285)
LM number, multiple	29/39 (0.296)	36/79 (0.012)	-
LM number, > 4	-	-	41/15 (0.597)
Bilaterality of LM, bilateral	50/31 (0.631)	100/61 (0.129)	36/0 (0.056)
Pretreatment CEA, > 5 ng/ml	41/27 (0.182)	57/68 (0.551)	29/23 (0.424)
Perioperative chemotherapy for LM	50/23 (0.067)	72/59 (0.171)	-
Surgical procedure, lobectomy	44/28 (0.537)	57/69 (0.980)	-
Use of molecular targeted drugs	-	-	35/26 (0.62)

CRC: colorectal cancer, LM: lung metastases, DFI: disease-free interval, CEA: carcinoembryonic antigen, DFS: disease-free survival, OS: overall survival

## Discussion

In this study, we investigated the survival outcomes of patients who underwent complete resection and those who received systemic chemotherapy for LM from CRC. The present study was limited to patients who began treatment for isolated LM after MTDs became available in Japan, as there has been no investigation regarding the impact of newer drugs on postoperative outcomes in this patient population. Despite a high recurrence rate, this study showed a favorable survival outcome of pulmonary metastasectomy. Multiple LM and rectal cancer were poor prognostic factors for OS and DFS in surgical patients, respectively. Rectal cancer predicted a poor prognosis for OS in chemotherapy patients.

The present study showed a favorable 5-year OS rate of 64.5% in patients treated with pulmonary metastasectomy for LM from CRC. However, tumor recurrence after complete resection occurred in more than half of the patients within five years. This suggests that treatment for recurrence after metastasectomy, such as repeated surgery and chemotherapy, would be important to improve the survival of these patients. Repeated surgery is considered an effective treatment [20], and six of the eight patients who underwent repeated surgery in our study did not have a second tumor recurrence without systemic chemotherapy after repeated surgery. However, this procedure is selective, being performed in patients with favorable physical and oncological conditions [21]. Thus, a substantial portion of patients who underwent metastasectomy would receive systemic chemotherapy for recurrence after metastasectomy [21]. Over the last decade, the 5-year survival of patients with pulmonary metastasectomy has rapidly improved since the advent of new active agents [13, 14]. Our result also showed significantly better survival than that of previous studies conducted before the introduction of PT, with survival rate of 32.4–45% [13, 22–25]. Therefore, PT-based agents are likely to prolong the survival outcome of pulmonary metastasectomy for LM from CRC. However, clinical impact of MTDs was uncertain, given the more favorable outcomes shown by several studies before the introduction of MTDs, with 5-year OS rate of 64.4– 71.8% [6, 13, 26, 27]. Although we could not conclude the efficacy of MTDs in this retrospective nature, our study could reflect actual clinical outcomes of pulmonary metastasectomy for LM from CRC after the introduction of MTDs.

To date, several factors have been reported to be determinants of poor surgical outcomes of LM from CRC, including multiple LM, rectal cancer, a short DFI, and increased serum carcinoembryonic antigen levels [3, 4, 28, 29]. The present study showed that multiple LM resulted in a significantly worse 5-year survival of 36% compared to that of a solitary metastasis in patients treated with pulmonary metastasectomy. Patients with multiple LM would be more likely to have occult micrometastases in the lung or extrapulmonary organ than solitary LM, and these micrometastases would result in an incomplete resection and associate with poor prognosis after surgery [4, 28]. In terms of DFS, rectal cancer was related to a poor prognosis in this study. Venous return from the rectum is via the iliac system, whereas venous return from the colon is primarily via the portal circulation. Thus, tumor spread from rectal cancers might be more systemic than from colonic cancers [29]. Moreover, the rectum has a richer lymphatic network than the colon, which may allow cancer cells to spread into the pelvic lymph nodes and increase the risk of recurrence [30]. If patients have these prognostic factors, close follow-up should be conducted for the earlier detection of tumor relapse after pulmonary metastasectomy.

In the present study, 5-year OS rate of patients treated with chemotherapy alone was 26.8%, with median OS time of 24.3 months. Our result contradicts the general assumption that the 5-year OS rate is almost zero without metastasectomy [15], which could be resulted from the development of systemic chemotherapy [13]. Compared with fluorouracil and leucovorin alone, the addition of irinotecan or oxaliplatin has extended median survival from 11 to 12 months to more than 20 months. In addition, the development of MTRs such as cetuximab or bevacizumab has led to further prognostic improvements in mCRC with median survival times of 28.7 months [8–12]. However, there is a lack of data on the efficacy of chemotherapy in treating isolated LM from resected CRC. To our knowledge, there is only one study reporting oncological outcomes of chemotherapy in unresectable isolated LM, which demonstrated the median OS time of 19 months and 2-year OS rate of 38.8% [31]. However, the previous study included only PT-based regimens as the first-line treatment. Therefore, our better survival outcome might reflect the efficacy of MTDs as the first-line of systemic chemotherapy for unresectable isolated LM, as well as other studies that showed the survival benefit of MTDs for mCRC [8–12]. Given the lack of literatures, our study could provide a good benchmark for survival rate of unresectable isolated LM from CRC in the modern systemic chemotherapy era.

Although a lot of literature has been published to evaluate the survival of LM, they focused on only patients who underwent pulmonary metastasectomy and there were currently few articles showing survival rates of both surgery and chemotherapy in a single paper. To our knowledge, only two studies have retrospectively compared the survival outcome of surgery to that of chemotherapy and/or best supportive care [32, 33]. However, these studies included many patients with unresectable extrapulmonary diseases, and MTDs were not used. Therefore, we restricted our study to a population of patients with isolated LM who had received aggressive chemotherapy, including PT and MTDs, to determine differences in survival in the age of modern chemotherapy. Although we recognized that there were selection mechanisms that favored surgical cases, the notable difference in 5-year survival rate with pulmonary resection could support the proposal that surgical resection could be considered as a valid treatment option for LM from CRC.

Our study had several limitations. First, our analysis was based on a small number of patients from one institution, making the study underpowered to identify any other true differences. Moreover, this small sample size made multivariate analysis inappropriate, and the present analysis cannot rule out the possibility of confounding factors. Second, the retrospective nature of this study may have led to the potential risk of bias, including selection biases. Specifically, patients in the chemotherapy group had more advanced lung metastases, as seen in the analysis comparing patient backgrounds in the surgery and chemotherapy groups. Third, two of the 72 patients underwent complete resection for extrapulmonary metastases after the initiation of LM treatment, which could interfere with the assessment of treatment outcomes for LM. Fourth, the median follow-up of 3.9 years for surgical patients and 2.0 years for chemotherapy patients may be too short to detect tumor recurrence and evaluate treatment outcomes. Given these limitations, our results should be interpreted with caution, and multicenter prospective studies with larger sample sizes are needed to validate our findings.

## Conclusions

Pulmonary metastasectomy showed a favorable long-term survival in patients with LM from CRC. Despite the high recurrence rate following metastasectomy and recent advancements in systemic chemotherapy, surgical resection could still be considered as a valid option among multidisciplinary treatments for LM from CRC.

## Data Availability

The datasets used and/or analyzed during the current study are available from the corresponding author on reasonable request.

## Abbreviations

CRC: colorectal cancer
LM: lung metastases
MTD: molecular targeted drug
BEV: bevacizumab
VEGF: vascular endothelial growth factor
NCCN: National Comprehensive Cancer Network
CT: computed tomography
DFI: disease-free interval
OS: overall survival
DFS: disease-free survival
PFS: progression-free survival
IRT: irinotecan
5-FU: fluorouracil
PT: platinum
PMAB: panitumumab
